# A Paradigm for Post-Covid-19 Fatigue Syndrome Analogous to ME/CFS

**DOI:** 10.3389/fneur.2021.701419

**Published:** 2021-08-02

**Authors:** Angus Mackay

**Affiliations:** The Brain Health Research Centre, University of Otago, Dunedin, New Zealand

**Keywords:** COVID-19, Post-COVID Fatigue Syndrome, ME/CFS, chronic fatigue syndrom, stressors, inflammatory mediator, hypothalamic paraventricular nucleus, neuroinflammation

## Abstract

A significant proportion of COVID-19 patients are suffering from prolonged Post-COVID-19 Fatigue Syndrome, with characteristics typically found in Myalgic Encephalomyelitis/Chronic Fatigue Syndrome (ME/CFS). However, no clear pathophysiological explanation, as yet, has been provided. A novel paradigm for a Post-COVID-19 Fatigue Syndrome is developed here from a recent unifying model for ME/CFS. Central to its rationale, SARS-CoV-2, in common with the triggers (viral and non-viral) of ME/CFS, is proposed to be a physiologically severe *stressor*, which could be targeting a *stress-integrator*, within the brain: the hypothalamic paraventricular nucleus (PVN). It is proposed that inflammatory mediators, released at the site of COVID-19 infection, would be transmitted as *stress-signals, via* humoral and neural pathways, which overwhelm this *stress-center*. In genetically susceptible people, an intrinsic *stress-threshold* is suggested to be exceeded causing ongoing dysfunction to the hypothalamic PVN's complex neurological circuitry. In this compromised state, the hypothalamic PVN might then be hyper-sensitive to a wide range of life's ongoing physiological *stressors*. This could result in the reported post-exertional malaise episodes and more severe relapses, in common with ME/CFS, that perpetuate an ongoing disease state. When a certain *stress-tolerance-level* is exceeded, the hypothalamic PVN can become an epicenter for microglia-induced activation and neuroinflammation, affecting the hypothalamus and its proximal limbic system, which would account for the range of reported ME/CFS-like symptoms. A model for Post-COVID-19 Fatigue Syndrome is provided to stimulate discussion and critical evaluation. Brain-scanning studies, incorporating increasingly sophisticated imaging technology should enable chronic neuroinflammation to be detected, even at a low level, in the finite detail required, thus helping to test this model, while advancing our understanding of Post-COVID-19 Fatigue Syndrome pathophysiology.

## Introduction

Around 10–30% of those 147 million people, worldwide (1), who have contracted *Corona Virus Disease 2019* (COVID-19), appear to be suffering ongoing, long-term effects of the disease, despite the virus, Severe Acute Respiratory Syndrome Coronavirus 2 (SARS-CoV-2), no longer being detectable (2, 3). These *post-COVID-19* patients appear to be a mix of those impacted directly from effects of the virus, itself, which have resulted in, for example, long-term pulmonary, cardiovascular and brain damage, as well as another substantial cohort, who independently, or comorbidly, have been experiencing ongoing “fatigue” or “post-viral fatigue,” as illustrated in these early reports (4–6). A multi-state telephone survey, in the United States, reported that 35% of 292 symptomatic adults (including one in five of those aged between 18 and 34 years, with no other chronic medical conditions) had not returned to their usual state of health 2–3 weeks after they had tested positive for COVID-19 (4). An Italian hospital study found 87% of their 143 *post COVID-19* acute-care patients surveyed, had not fully recovered 2 months after admission, while reporting ongoing “fatigue” (5). An Irish study of 128 *post-COVID-19* patients, who had recovered from the acute phase of COVID-19 infection (half required hospital admission), found 52% reported persistent “post-viral fatigue” at a median of 10 weeks after initial COVID-19 symptoms (6), and of those 31% were unable to return to work.

As the pandemic has progressed increasing concern has been raised that the loosely defined, but widespread “post-viral fatigue” might in fact represent a much more entrenched and serious form of SARS-CoV-2 triggered “post-viral fatigue syndrome,” in a significant proportion of this cohort (2, 3). Post-Viral Fatigue Syndrome (PVFS) is essentially the same disease as Myalgic Encephalomyelitis/Chronic Fatigue Syndrome (ME/CFS), originally named simply as Myalgic Encephalomyelitis (ME) after it resulted from an infectious disease outbreak at the Royal Free Hospital in London, in 1955 (7). The World Health Organization lists PVFS and ME/CFS under the same category of neurological disorders (8), while clinical (diagnostic) assessments are the same for each disease. ME/CFS-like illnesses have arisen mainly from a “post-viral fatigue” documented as a consequence of over 75 reported localized outbreaks of infectious disease, since the 1930's (9), but all dwarfed by the pandemic of COVID-19. However, unlike PVFS, the triggers of ME/CFS can sometimes be non-viral (10), which may explain why both terms persist.

Fauci noted that some *post-COVID-19* patients' symptoms, such as difficulty in concentrating (“brain fog”) and “fatigue,” were highly suggestive of ME/CFS (3). A UK study estimated that 10% of its patients who had tested positive for COVID-19 remained unwell more than 3 weeks later, and a smaller proportion months later (11). It stated: “The profound and prolonged nature of fatigue in some post-acute COVID-19 patients shares features with *chronic fatigue syndrome*, described after other serious infections including SARS, MERS, and community acquired pneumonia.” The Irish study, mentioned earlier, also reported a gender bias with 67% of those suffering from “post-viral fatigue” being female (6); ME/CFS has a significant gender bias toward females (10).

Significantly, “chronic fatigue” lasting 6 months or longer, without an alternative explanation, is required to fulfill a diagnosis of ME/CFS (10). Longitudinal studies tracking *post-COVID-19* patients' symptoms over 6 months or more, are only now emerging, as COVID-19 is still a relatively new, and complex disease, with multiple outcomes. Increasing links between *post-COVID-19* patients' symptoms and ME/CFS are becoming evident, as the three recently completed longitudinal studies, following, illustrate.

A Chinese (Wuhan) study of a large patient cohort (*n* = 1,733), discharged from hospital, found that 76% of them had at least one persistent symptom 6 months after their initial COVID-19 onset (12). The most common symptoms reported were fatigue or muscle weakness (63%), sleep difficulties (26%) and anxiety or depression (23%), which are common in ME/CFS (8). Of the patient cohort, 75% had required supplemental oxygen, with 7% on a ventilator, during their hospital stay, whereas the remaining 25% did not. Consequently, impaired pulmonary diffusion capacity and abnormal chest imaging manifestations were reported (estimated at 22–56% across a scale of severity, and especially evident in the more severe cases). This could also have contributed to the specific symptoms reported in a subset of patients.

An international, web-based survey involved 3,762 participants, aged between 30 and 59 years old (13). Of those with *post-COVID-19* issues, 6 months since their infection, 80% were female. The most frequently reported symptoms were fatigue (>75%), post-exertional malaise (>69%) and cognitive dysfunction (“brain-fog”) (>52%), all common core symptoms of ME/CFS. Over 85% experienced health relapses, induced by physical, mental exercise or psychological stress, also key characteristics of ME/CFS (6). Around 67% of the cohort were unable to work or were on a reduced work schedule at the time of the survey. Most of those surveyed (>90%) had not been hospitalized indicating that even relatively mild cases of SARS-CoV-2 infections could trigger ME/CFS-like symptoms.

A comprehensive German study of 42 *post-COVID-19* patients, aged between 22 and 62 years old, of which 29 were female, is the first to assess a *post-COVID-19* cohort clinically for ME/CFS (14). They were assessed 6 months after initial SARS-CoV-2 infection, which had caused only mild to moderate COVID-19 induced symptoms, thus ruling out interpretations of their long-term symptoms that might be relevant in more severe (hospitalized) cases of COVID-19. The most frequently reported ME/CFS symptoms were chronic fatigue by all 42 patients, post exertional malaise (PEM) (*n* = 41), cognitive impairment (*n* = 40), headache (*n* = 38), and muscle pain (*n* = 35). The 2003 Canadian Consensus Criteria were the ME/CFS diagnostic criteria used. These were met by 19/42 patients, who were diagnosed with severe fatigue and cognitive impairment, severe stress intolerance, and hypersensitivity to noise, light, and temperature. In particular, the intensity and duration of PEM (symptoms lasting for more than 14 h) was considered to be the main diagnostic criterion for ME/CFS. The remainder of the patients (*n* = 23), in the study, shared many of these same symptoms, but they were at a lesser severity (in particular PEM duration lasted between 2 and 10 h), and they were categorized as having a closely related “Chronic COVID-19 Syndrome.” This study, again, illustrated that even mildly affected *post-COVID-19* patients can develop ME/CFS-like symptoms. The authors concluded: “Our study provides evidence that patients following mild COVID-19 develop a chronic syndrome fulfilling diagnostic criteria of ME/CFS, in a subset. We must anticipate that this pandemic has the potential to dramatically increase numbers of ME/CFS patients.”

As this study has typified, there is currently a lack of consensus on what to name this debilitating *post-COVID-19* illness, with its possible subtypes (15). Therefore, for the purposes of the remainder of this article, an all-encompassing term, “Post-COVID-19 Fatigue Syndrome,” will be used. As yet, no clear pathophysiological explanation for any such type of Post-COVID-19 Fatigue Syndrome (PCFS) has been proposed. Due to its similarity with ME/CFS, a novel paradigm for PCFS is developed here from a recent unifying model of ME/CFS (16, 17). Integral to this paradigm a unique rationale to explain how SARS-CoV-2 infection might be triggering PCFS, in common with the known triggers of ME/CFS is presented (10). Then the paradigm is developed to explain post-exertional malaise (PEM), and the longer-term relapses, being reported in PCFS, both of which are key characteristics of ME/CFS (10). Other symptoms common to PCFS and ME/CFS are also explained, before a model for PCFS is provided, along with some suggestions as to how it might be validated.

## Rationale for How SARS-CoV-2 Triggers Post-COVID-19 Fatigue Syndrome (PCFS)

The rationale is based around the premise that since PCFS has many ME/CFS-like characteristics, a common triggering mechanism for both syndromes is plausible. ME/CFS, by contrast, however, is known to have many different wide-ranging triggers apart from infectious diseases, such as vaccinations, chemical toxins and emotional trauma (10). The key question is whether there is a common determining factor shared by all of these triggers in the mechanism of onset of ME/CFS, and by its close association, PCFS.

The authors of an Australian study (2006), which followed the progression of three different debilitating infectious diseases, each known to cause ME/CFS, also posed questions about what might be triggering ME/CFS (18). The sample of 253 patients studied had been infected by quite distinct pathogenic potential triggers of ME/CFS—Ross River Virus, an RNA virus that targets the joints; Epstein-Barr Virus, a DNA virus that causes infectious mononucleosis and targets B-lymphocytes; and Q fever, caused by a rickettsia bacterium. However, each triggered ME/CFS in proportionally the same number of patients (around 12% of those infected), and with similar symptom characteristics. Interestingly, the pathophysiology of SARS-CoV-2 is also quite distinct from these agents—it is a novel RNA coronavirus, which initially invades host cells in the respiratory tract, but whose effects can be widespread (19, 20). Although distinct in their individual pathogenic outcomes, each disease would likely produce a similar severe “inflammatory response,” at the site of infection. Might the traumatic effect of these individual infections, each acting as a severe physiological *stressor* associated with their mutually intense “inflammatory responses,” be the common determining factor in their triggering mechanism?

Likewise, the other triggers of ME/CFS (10), such as emotional trauma, multiple vaccinations (that can also precipitate a significant “inflammatory response”) and chemical toxin “shock” share the same trait—that of being a severe physiological *stressor*. Of interest, Gulf War Illness, which affected around 250,000 USA soldiers from the Gulf War (1990/1991), presented with ME/CFS-like symptoms (21). Those affected were exposed to a powerful concoction of physiological *stressors via* multiple vaccinations and chemical toxin exposures, potentially coupled to war-induced post-traumatic stress disorder. A normal human physiological response, menopause, can be particularly physiologically stressful in some women, and it has also been acknowledged as a potential trigger of ME/CFS (22).

It is proposed, therefore, that SARS-CoV-2 infection, in common with the triggers of ME/CFS, manifests itself as a severe physiological *stressor*. An essential target could be the brain's stress-center, a cluster of neurons in the hypothalamic paraventricular nucleus (PVN), which has been proposed previously to be the target site in ME/CFS (16, 17). The hypothalamic PVN is a complex array of nuclei and neurological circuitry, which functions as a *stress-integrator*, absorbing, processing and responding to a wide range of physiological stressors, playing an essential role in neuroendocrine and autonomic regulation (23, 24). Incoming *stress-signals* from all types of infections (via inflammatory mediators, such as cytokines and chemokines), pain, emotional distress and cardiovascular changes from physical exertion, all converge upon the hypothalamic PVN, by a range of humoral and neural routes. This single point of convergence, the hypothalamic PVN, is a potential vulnerable site in genetically susceptible people. The Australian study (18), which studied the development of ME/CFS from three quite distinct pathogenic triggers, indicated that ~1 in 10 might have some kind of genetic susceptibility. If that ratio was applied to SARS-CoV-2 infection, then those estimated to have PCFS would be in the order of tens of millions of people, worldwide, and presents a significant ongoing global health problem. The hypothalamic PVN, could be at the heart of the pathophysiological mechanism, as proposed here.

SARS-CoV-2 has been shown to provoke a typical inflammatory response, at the initial site of infection in the lungs, leading to a surge in the release of chemokines and pro-inflammatory cytokines such as IL-6, TNF, and IL-1β (19). Studies indicate that the more severe the COVID-19 infection, the greater the *cytokine storm* that ensues (leading potentially to the development of Acute Respiratory Distress Syndrome) (19, 20). Pro-inflammatory cytokines (*stressors*) released into systemic circulation act on circumventricular organs—areas of the brain that have incomplete blood-brain barriers and can therefore detect chemical signals, for example, the area postrema, which then relays these as *stress-signals* onto the hypothalamic PVN (23). Likewise, cytokines (*stressors*) released by immune cells in lymphoid tissue also communicate to the (ascending) vagus nerve that an inflammatory process is occurring. The vagus nerve is connected to another circumventricular organ, the nucleus tractus solitarius, which also relays inflammatory mediators, as *stress-signals*, onto the hypothalamic PVN. Raised levels of these inflammatory mediators (*stressors*), therefore, cause an influx of *stress signals* targeting the hypothalamic PVN (20, 21). However, the previously discussed Irish study found that there was no correlation between the severity of the initial SARS-CoV-2 infection (as was indicated by blood-levels of pro-inflammatory and immune cell markers, during the acute phase of COVID-19 infection), with those who experienced ongoing “post-viral fatigue” (6). As the 128 patients in this study were a mixture of non-hospitalized and hospitalized cases it confirms that relatively mild cases of COVID-19 can cause “post-viral fatigue,” leading potentially to PCFS.

**Figure 1:**
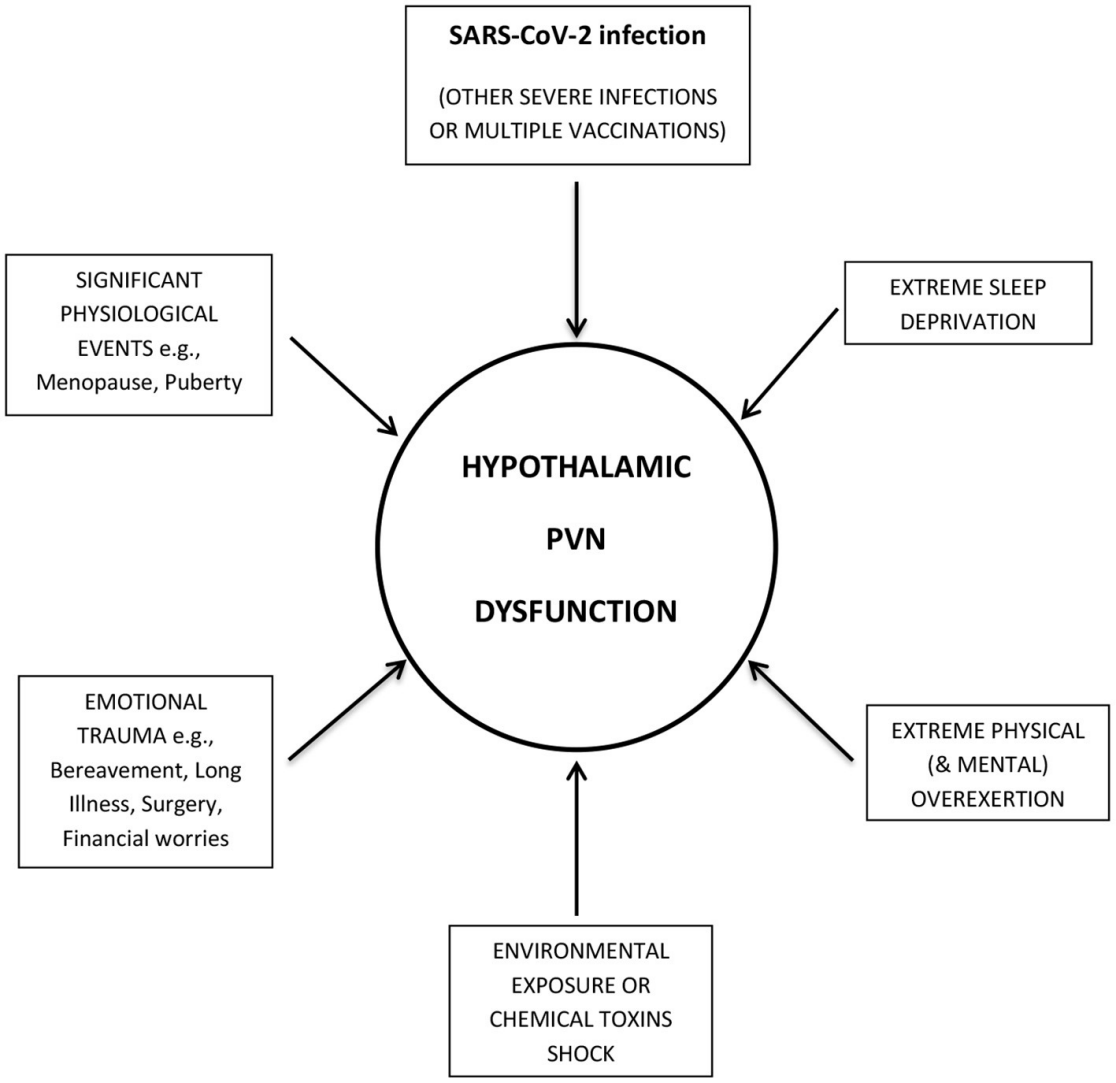
SARS-CoV-2 infection, in common with other *stressors*, targets the Hypothalamic Paraventricular Nucleus (PVN). SARS-CoV-2 infection produces an inflammatory response, which, in common with other physiological *stressors* (and potential triggers of ME/CFS), targets the hypothalamic paraventricular nucleus (PVN). If a certain *stress*-*threshold*, intrinsic to genetically susceptible people, is exceeded, the hypothalamic PVN switches into an ongoing dysfunctional mode, causing Post-COVID-19 Fatigue Syndrome.

Therefore, it is proposed that a SARS-CoV-2 infection, in common with other physiological *stressors* [and potential triggers of ME/CFS (18, 19)], is able to switch the hypothalamic PVN, into an ongoing dysfunctional mode (Figure 1). Even a relatively mild SARS-CoV-2 infection would appear to be able to provoke a sufficiently large enough inflammatory response (*stressor*), in some patients, to trigger PCFS. Perhaps an intrinsic *stress*-*threshold* particular to the hypothalamic PVN is lower in genetically susceptible individuals, and if exceeded, is integral to its pathophysiological mechanism. In many cases, and perhaps in even milder cases of COVID-19, the triggering event might require the cumulative action of multiple *stressors* to exceed the *stress*-*threshold*. This cumulative effect could also help explain why ME/CFS patients are often unable to identify a specific triggering event for the onset of illness (10).

## Post-Exertional Malaise and Relapses in PCFS Explained

As outlined earlier, more recent studies of *post-COVID-19* patients, over an extended period (6 months or more), have highlighted post-exertional malaise (PEM) and relapses (13, 14), both key characteristics of ME/CFS (10). Also, in common with the many triggers of ME/CFS, these set-backs seem to occur despite the triggering event (SARS-CoV-2, in the case of PCFS), no longer being current or detectable. So, how might PEM and relapses be explained in PCFS? Instructively, ME/CFS patients are known to be intolerant to a wide range of “life's” normal physiological *stressors*. These encompass physical or mental overexertion, emotional or financial stress, sleep deprivation, and environmental *stressors*, such as exposure to chemical toxins, vaccinations and infections, or ingestion of alcohol (10). This would appear to be the case, also, in PCFS (13, 14). Interestingly, these *stressors* are similar in type and diversity (if not always in severity), as those which are known to trigger ME/CFS. Therefore, they would likely mirror their action, using the same humoral and neural routes, to target a now compromised and stress-sensitive hypothalamic PVN (and as illustrated in Figure 1). When a certain (but variable) *stress-tolerance-level* is exceeded, depending on the severity and duration of the trauma, short-term PEM, medium-term “flare-ups,” or longer-term relapses would eventuate.

## Symptoms of PCFS Explained

**Figure 2:**
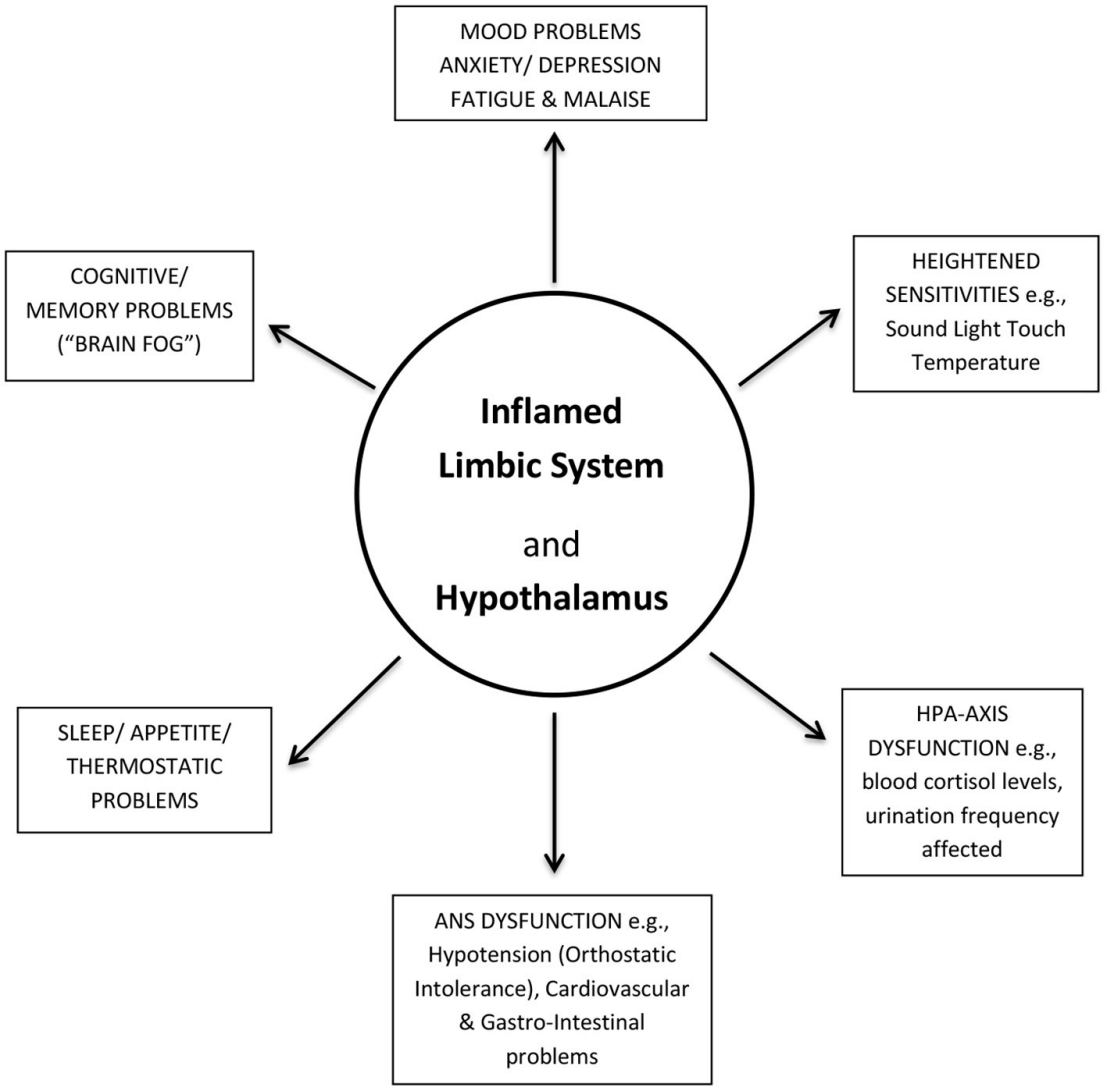
Post-COVID-19 Fatigue Syndrome (PCFS) symptoms explained. An inflamed (dysfunctional) limbic system and its hypothalamus could account for the majority of the wide-range of ME/CFS-like symptoms in PCFS. ANS, Autonomic Nervous System; HPA-axis, Hypothalamic-Pituitary-Adrenal axis.

I propose that a dysfunctional, overloaded hypothalamic PVN, can act as an epicenter for localized microglia (and astrocyte) induced activation. Pro-inflammatory cytokines and neurotoxic molecules would be released in response by microglial and astrocyte cells (the innate immune cells of the brain), causing neuroinflammation to spread throughout the hypothalamus and into its proximal, closely connected, limbic system. A dysfunctional hypothalamus and limbic system could then explain the majority of the wide range of ME/CFS-like symptoms being reported in PCFS (Figure 2) (11, 12), and as has been explained in more detail previously for ME/CFS (18, 19).

Since PCFS has yet to be investigated for neuroinflammation in the brain, evidence pertaining to such an innate immune (inflammatory) response is drawn from a few key ME/CFS studies. Activated microglia and astrocytes have been detected within the limbic system (cingulate cortex, hippocampus, amygdala, and thalamus) of ME/CFS patients in a seminal Japanese Positron Emission Tomography/Magnetic Resonance Imaging (PET/MRI) study, of 2014 (25). The nearby mid-brain and pons region (in the brain-stem) were also identified as being potentially affected. A commonly used ligand (^11^C-R-PK11195) for a translocator protein (TSPO), which is expressed by activated microglia and astrocytes, provided an indicator of ongoing neuro-inflammation in the study. The severity of symptoms self-reported, such as fatigue, cognitive impairment, pain and depression, correlated with the intensity of their brain glial-cell activation. The hypothalamus was not highlighted specifically in this study, but a more recent MRI study which correlated autonomic dysfunction, measured by heart-rates and blood-pressure readings, for ME/CFS patients and healthy controls found abnormalities in the brain stem and hypothalamus, itself (26). Interestingly, another recent MRI study indicated that post-exertional malaise (PEM) could be correlated to a reduction in cognitive function of ME/CFS patients, but with increased brain activity, which was detected within the inferior and superior parietal and cingulate cortices—a region of the limbic system (27). A recent Magnetic Resonance Spectroscopy (MRS)/ MRI thermometry study, also highlighted significantly raised lactate levels (indicative of mitochondrial stress) and raised temperature levels (indicative of inflammation) in parts of the limbic system (anterior cingulate cortex and thalamus) of ME/CFS patients (28). Mitochondrial dysfunction and inflammation within the brains of ME/CFS patients has also been implicated in several recent cerebral spinal fluid biomarker studies (29–31). However, it is not clear if mitochondrial dysfunction might be an effect, an intermediary cause of the neuroinflammation, or both.

## A Model for Post-COVID-19 Fatigue Syndrome (PCFS)

**Figure 3:**
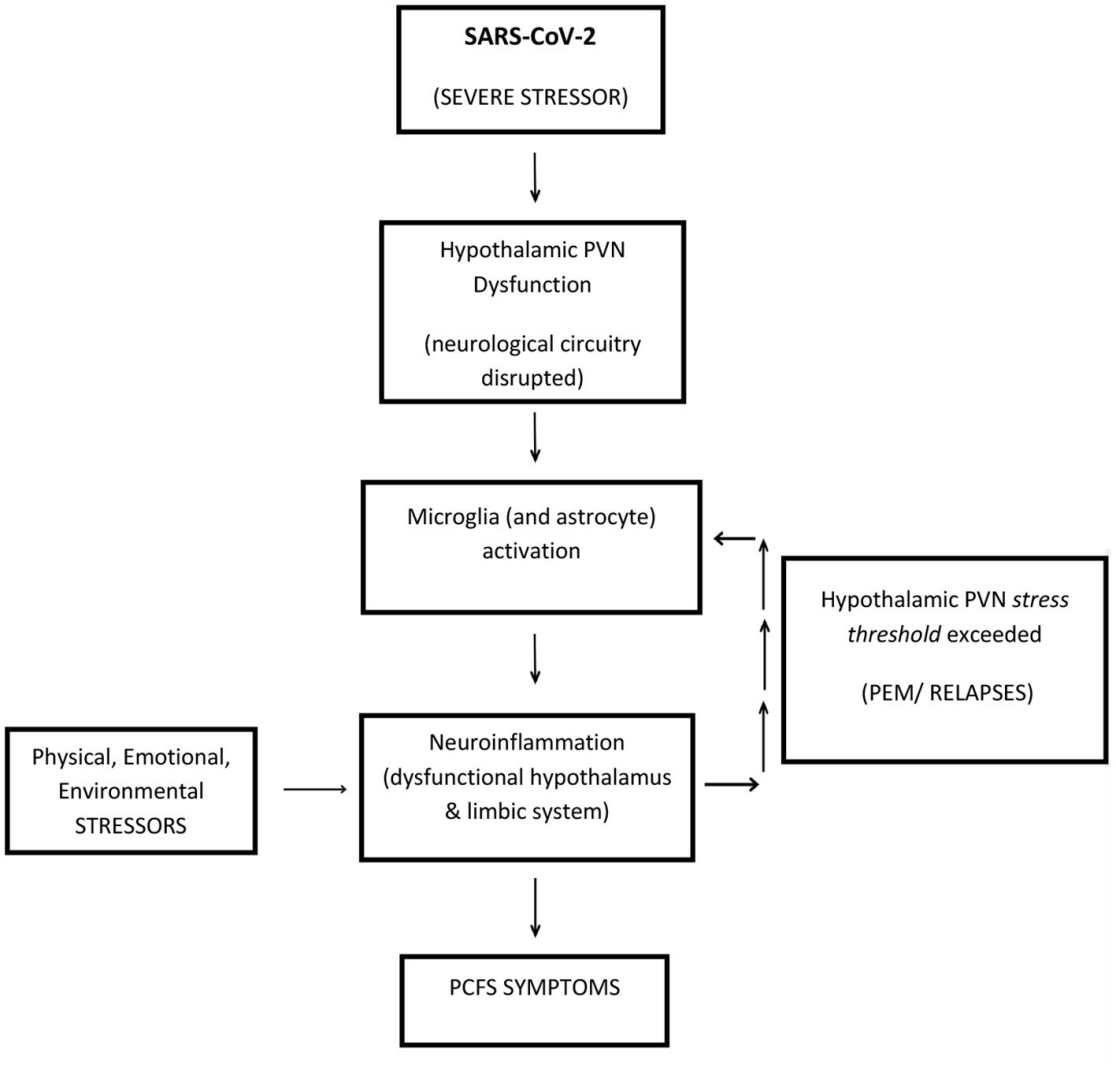
A neuroinflammatory model to explain Post-COVID-19 Fatigue Syndrome (PCFS). SARS-CoV-2 presents as a physiologically SEVERE STRESSOR, which targets the hypothalamic PVN, in genetically susceptible people. The hypothalamic PVN is traumatized into a permanently dysfunctional mode. Ongoing (life) STRESSORS continue to target a now compromised *stress-sensitive* hypothalamic PVN, causing the perpetuation of PCFS *via* short-term PEM, or longer-term RELAPSES. Accordingly, a dysfunctional hypothalamic PVN acts as an epicenter to neuroinflammation radiating outwards *via* localized microglia-induced activation. Resultant dysfunction of the hypothalamus and its surrounding limbic system could account for the majority of symptoms of PCFS. This figure is a modified version of Figure 1 in the study by (19) and Figure 1 in the study by (20).

A neuroinflammatory model is presented, below (Figure 3), for PCFS, to stimulate discussion and provide a framework for future testing and critical evaluation. Central to its reasoning it categorizes SARS-CoV-2 infection, as a physiologically severe *stressor*, which targets a potentially vulnerable site (in genetically susceptible people)—the hypothalamic PVN.

It is simple and coherent as it proposes that ongoing physiological *stressors*, responsible for perpetuating PCFS do so by insulting the same target—a now compromised and *stress*-*sensitive* hypothalamic PVN. These *stressors* then become the “drivers” of the disease sustaining the inflammatory process indefinitely, as characterized by recurrent PEM set-backs and relapses. The hypothalamic PVN is optimally positioned to be at the epicenter of PCFS pathophysiology where microglial-induced neuroinflammation affects both the key-functioning hypothalamus and its proximal, closely connected limbic system, which can then explain the diverse range of ME/CFS-like symptoms in PCFS. This model for SARS-CoV-2 triggered PCFS is applicable to all the triggers of ME/CFS—it is a unifying model (16, 17). Interestingly, the hypothalamic PVN is a vulnerable point in other conditions related to hypertension and congestive heart failure, where neuroinflammation of the hypothalamic PVN, *via* activated glial cells, is implicated (24, 32).

## Discussion

This section suggests how this model might best be validated, and while furthering our understanding of PCFS pathophysiology it should also advance our understanding of ME/CFS.

Of fundamental significance, since the brain is devoid of nociceptors (33), there is no sensation of pain (and heat) that one might expect from inflammation, as proposed here. PCFS patients might report the causal effects of any inflammation, as described earlier (13, 14), such as cognitive difficulties (“brain-fog”) and mood problems, but not the inflammation itself. The standard diagnostic tests available to any clinician, nor those used in general for brain-scanning studies, until recently, have not been sensitive enough to detect any such inflammation. Without a definitive root source to explain their symptoms (as described in Figure 2) the medical community has been left perplexed about the true pathophysiological basis of PCFS, as they have been about ME/CFS, since its inception (34). The key Japanese PET/MRI study of 2014 showed, however, that with more advanced technology the chronic, but low-level (variable) neuroinflammation, likely present in PCFS, is detectable, and with the precision required. Regrettably, to date, their “breakthrough” study has not been reproduced by any other research study. However, the rapid development of new and more sensitive PET/MRI tracers (and the receptors, which they target) has continued, and gives hope that any such future studies, be they into PCFS, or ME/CFS will be greatly enhanced (35). Such hope is reflected through the ongoing PET/MRI studies of ME/CFS patients, by the Japanese group (36). They report that they have successfully had their earlier study ratified (in collaboration with the Karolinska Institute), using a new PET ligand, [^11^C] PBR-28. They have also completed an expanded repeat study (60+ ME/CFS patients), using a newly developed PET ligand, [^18^F] DPA-714 (unpublished). Senior author, Watanabe reports: “neuroinflammation is in some level in the hypothalamus, too, but because of the problem of spatial resolution, we could not say PVN or other hypothalamic nuclei…” This report further emphasizes the urgent need for more brain-focused studies, using yet more sensitive and advanced PET/MRI technology, with the ultimate goal of providing a *brain*-*signature* for PCFS. Careful comparisons of neuroinflammatory images or “maps” can then be made between PCFS and ME/CFS to validate their identities. Comparisons could also be made with other potential neuroinflammatory diseases, such as fibromyalgia, epilepsy, migraines and mental health disorders. A recent PET/MRI study of the closely-related fibromyalgia provided evidence that individual *brain-signatures* are achievable (37), as glial-cell activity in a different region, the cortex was elevated relative to healthy controls. However, in common with the Japanese ME/CFS PET/MRI study (25), as one might expect for such a closely-related disease, there was some overlap of inflammation found within the limbic system, where activated glial-cells were detected within the anterior and posterior middle cingulate cortices.

A golden opportunity for large-scale, longitudinal, brain-imaging studies of *post-COVID-19* patients displaying ME/CFS-like symptoms, to enable better understanding of PCFS disease progression through relapse-recovery cycles has been presented to the scientific community by this pandemic. Such studies carried out in tandem with the recordings of patients' symptoms, in addition to cerebral spinal fluid (CSF) and blood biomarker data will help to build a more in-depth understanding of its pathophysiology. Such studies might be complicated by the systemic organ damage caused by COVID-19 in more severe cases. Therefore, it might be sensible to study first *post-COVID-19* patients with milder (non-hospitalized) cases of COVID-19, as other studies mentioned earlier have done (13, 14). This will help to rule out other possible overlapping causes of symptoms, from more severe COVID-19 cases, such as: post intensive care syndrome, pulmonary impairment, neurological deficits and posttraumatic stress disorder (14).

In the absence of an animal model, brain-tissue studies might help elucidate the mechanistic detail of the neuroinflammatory pathway, within the brains of PCFS patients. It might conceivably follow the same neuroinflammatory pathway, which is reported to be shared by “early stage” neurodegenerative (progressive neuroinflammatory) diseases (38), such as Alzheimer's Disease (AD), Parkinson's Disease (PD), amyotrophic lateral sclerosis (ALS) and multiple sclerosis (MS), and as has been suggested previously for ME/CFS (23, 24). Common to each, in its initial onset, is the activation of microglia and astrocytes. This is amplified by downstream signal transduction pathways present in microglia and astrocytes, resulting in the production of molecules, such as pro-inflammatory cytokines TNF-α, IL-1β, and IL-6, as well as neurotoxic reactive oxygen species (ROS), and nitric oxide (NO) (38–40). These may cause further amplification of the immune response, as well as neuro-inflammation and neuronal damage, and become progressively more damaging if the process continues unabated. ATP released by necrotic neurones, during apoptosis, might also contribute to this process by further activation of microglia through positive feedback loops. Both microglia and astrocytes possess purinergic receptors that can bind ATP present in the interstitial fluid. Microglia when activated can also release the neuro-transmitter glutamate in a sustained form which is thought to have a deleterious effect on neuronal mitochondria (mitochondrial respiratory chain complex IV becomes inhibited) (41). Mitochondrial dysfunction then could be a key component in the neuro-inflammatory process and account for the reports of lactate, produced by anaerobic respiration, as mentioned earlier being detected in the brains (28) and CSF of ME/CFS patients (29).

However, what appears to differentiate the pathological outcomes, of the neurodegenerative diseases, are the presence of disease-specific “endogenous factors,”' which trigger the inflammatory processes: *amyloid*-*beta* for AD, α-*synuclein* for PD, *mutant SOD1* for ALS, and *myelin*-*peptide*-*mimetic* for MS (38). The “endogenous factors” become the “drivers,” which sustain the inflammatory process. What might be the “driver” of the inflammatory process in PCFS (and ME/CFS)? Any such “endogenous factor” for a non-progressive neuroinflammatory disease, like PCFS, is likely to be more subtle, and therefore less conspicuous than the aggregated proteins, such as *amyloid-beta* or *alpha-synuclein* in progressive neuroinflammatory (neurodegenerative) diseases AD and PD, respectively. Possible candidates for ME/CFS, as have been suggested before (16, 17), and therefore applicable to PCFS, could be fragments of a virus, or a latent intact virus in the brains of patients, which is activated by stress, or be damaged mitochondria also linked to stress. Both of these potential “endogenous factors” could then stimulate microglia into activity, and drive the inflammatory process. However, it could be still more subtle, and undetectable in PCFS (and ME/CFS), if, as outlined earlier, the “drivers,” which sustain the inflammatory process are in fact “life's” normal physiological *stressors*, acting in tandem with a compromised *stress-sensitive* hypothalamic PVN. The *stressors*, which drive the microglia-induced inflammation would vary in their severity, which is mirrored by their effects—PEM or relapses, when a certain *stress-tolerance level* is exceeded. This too could be reflected by the degree of inflammation in the hypothalamus and the limbic system, thus accounting for the severity of the symptoms, at any one time.

Then, what might be the “missing-link” that links a “distressed” hypothalamic PVN with microglia-induced activation, if not viral or damaged mitochondrial intermediates? Interestingly, stress induces the release of corticotropin-releasing hormone (CRH) from the paraventricular nucleus of the hypothalamus. CRH activates glial-cells and mast cells through CRH receptors and releases neuroinflammatory mediators (42) Mast cells, resident to the brain, are linked to a broad range of neurodegenerative diseases and neuronal disorders such as depression, migraines and autism, likely play a part in the neuro-inflammatory process, and influence “blood-brain-barrier” permeability. Microglia and astrocytes may also respond to inflammatory mediators released by mast cells (43). Another possibility requiring further scrutiny is disruption to a potentially hypothalamic PVN-induced, *neural-glial crosstalk* mechanism, which is also relevant to neurodegenerative diseases (44), and that might be pertinent to PCFS (and ME/CFS). Interest has been intensifying upon a chemokine intermediate, *fractalkine*, and its receptor present on glial-cells, which could be disrupted in ME/CFS patients, thus inducing glial-cell activation (45). Intriguingly, a Swedish company has been planning a Phase 2 trial for a drug termed a *fractalkine receptor inhibitor* to prevent hyperinflammation in COVID-19 patients, and thereby Acute Respiratory Distress Syndrome (46). Such a drug might have relevance to PCFS (and indeed ME/CFS), if the dampening down of glial-cell activity is found to be beneficial to its therapy. Other drugs, which warrant further investigation, and arguably, clinical trials, are low-dose naltrexone (LDN) and Aripiprazole. LDN, a drug, which at low doses (0.5–4.5 mg/days) is proposed to have anti-inflammatory properties, has shown favorable results in fibromyalgia treatment (47), and in ME/CFS treatment, as three case studies have indicated (48). The authors write: “This series of three case reports compiled by people with long-term ill-health due to chronic fatigue syndrome shows the range of responses they observed when taking LDN, from life changing to a reduction in some symptoms only.” In a retrospective study the medical records of 101 patients who met the diagnostic criteria for ME/CFS, and who received off-label aripiprazole were reviewed. Of the 101 patients taking aripiprazole, 75/101 (74%) experienced an improvement in one or more categories of fatigue, brain fog, unrefreshing sleep, and frequency of post-exertional malaise episodes. The authors wrote: “… a growing body of evidence suggests that ME/CFS involves inflammation of the brain… dopamine D2 receptor agonists have been shown to mediate neuroinflammation, microglial activation… this suggests that dopamine-modulating drugs like aripiprazole may lead to clinical improvement in fatigue and cognitive symptoms in ME/CFS…” (49).

With over 147 million confirmed cases of COVID-19 worldwide currently (1), and globally still with an upward trend of a fourth wave of infections, the number of *post-COVID-19* patients with ME/CFS-like symptoms is likely to be in the order of millions. This is based on the most conservative of projections, and will present a globally significant ongoing health problem (50). With this impending flow on health crisis, it is hoped much needed funding will be forthcoming for more intensive research into PCFS, and also provide benefit for the estimated 15–20 million ME/CFS patients worldwide. A specific focus, on study of these illnesses as neurologically-based, stress-related, neuroinflammatory diseases will likely bring significant advances.

## Data Availability Statement

The original contributions presented in the study are included in the article/supplementary material, further inquiries can be directed to the corresponding author/s.

## Author Contributions

AM was the sole author, whom has conceived, prepared, and enabled a review process of the initial draft, before submitting the paper.

## Conflict of Interest

The author declares that the research was conducted in the absence of any commercial or financial relationships that could be construed as a potential conflict of interest.

## Publisher's Note

All claims expressed in this article are solely those of the authors and do not necessarily represent those of their affiliated organizations, or those of the publisher, the editors and the reviewers. Any product that may be evaluated in this article, or claim that may be made by its manufacturer, is not guaranteed or endorsed by the publisher.

## Abbreviations

PCFS: Post-COVID-19 Fatigue Syndrome.
